# Association of parental rearing styles with suicidal ideation in Chinese adolescent patients with depression: a large-scale cross-sectional study

**DOI:** 10.3389/fpsyt.2024.1414887

**Published:** 2025-01-09

**Authors:** Jiacheng Liu, Chang Cheng, Kamila Edeleva, Zhen Zhao, Liying Yang, Chuanyi Kang, Xiaohong Wang, Na Zhao, Jian Hu

**Affiliations:** ^1^ Department of Psychiatry, The First Affiliated Hospital of Harbin Medical University, Heilongjiang, Harbin, China; ^2^ Key Laboratory of Acoustic, Optical and Electromagnetic Diagnosis and Treatment of Cardiovascular Diseases, Heilongjiang, Haibin, China

**Keywords:** adolescent, depression, parental rearing style, risk factor, suicidal

## Abstract

**Background:**

Suicide is the first cause of death among adolescents globally and has a severe impact on socioeconomic development. Several studies have found that suicide attempts and suicidal ideation (SI) are more likely to occur in adolescents with depression. Previous studies have found that stressful events in early childhood, especially family stress, can influence the occurrence of SI. Chinese parents tend to be more strict and less able to express their emotions, owing to unique national conditions, which may result in more parenting problems. Therefore, our study investigates the risk factors for SI in a large sample of Chinese adolescents with depression.

**Methods:**

A total of 1604 adolescent patients with depression were recruited in this study. A self-administered questionnaire collected the clinical and demographic data. SI was assessed by interview. The Egna Minnen Beträffande Uppfostran (EMBU) scale was used to evaluate parental rearing style.

**Results:**

The study showed that female (OR=1.886, 95%CI:1.502–2.368), sleep (OR=0.798, 95%CI:0.637–0.998), school management (OR=1.179, 95%CI:1.041–1.336), alcohol consumption (OR=1.798, 95%CI:1.304–2.479), child life (OR=1.797, 95%CI:1.457–2.216), maternal interference (OR=1.032, 95%CI:1.015–1.048), paternal emotional warmth (OR=0.975, 95%CI:0.966–0.983) and paternal rejection (OR=1.102, 95%CI:1.063–1.142) were significantly associated with SI.

**Conclusions:**

To recognize SI earlier, physicians and nurses need to pay more attention to those female adolescent depression populations that are experiencing an unhappy childhood, non-democratic school management, alcohol consumption, excessive maternal interference, lack of paternal emotional warmth, and paternal rejection.

## 1 Introduction

Suicide is the leading cause of death among young people worldwide. More than 800,000 people die by suicide every year globally, which is the second-leading cause of death in U.S. adolescents (1). Adolescent suicide has become a widespread concern worldwide, and suicidal ideation (SI) is an influential signaling factor in predicting the occurrence of suicidal behavior in individuals. Suicide accounts for 8.5% of the total deaths among adolescents worldwide, which is the first cause of death (2). Suicide has also become the second leading cause of death among Chinese adolescents and has gradually increased in recent years (3). Among the nearly 80,000 people who die by suicide in China every year, about 3,000 are under 20 years old, and on average, one teenager dies by suicide every 3 hours (4). Although the suicide rate of adolescents is lower than that of adults, suicide can have a severe impact on the physical and mental health of adolescents as well as on the economy of their families and societies. Adolescence is the first stage in which a significant increase in the rate of suicide in the population is evident, and it is the first peak in the urban areas of the country (5). SI and suicidal attempts in adults also often begin in adolescence (3). The study of protective factors and risk factors associated with adolescent suicide plays a vital role in improving the living environment of adolescents and preventing suicide.

Suicide is a fatal self-injurious behavior caused by a combination of external circumstances and one’s situation. It starts with the development of SI, i.e., suicidal thoughts. It gradually progresses to a planned suicide attempt and finally to suicidal behavior. It is of great significance to explore the protective factors and risk factors related to adolescent suicide to improve adolescent mental health problems and prevent adolescent suicide in an effective and targeted manner. In recent years, many scholars have studied the factors associated with SI in adolescents, including sociocultural, psychological, biological, and environmental factors (6). However, the results of the studies obtained are not consistent. Some studies have found that a number of demographic factors may be associated with SI among adolescents, including being female, having fewer years of education, older age, living in rural areas, and low socioeconomic status of parents (7–9). In terms of family, adolescents with poor family structure (e.g., single-parent families, restructured families, and families with children left behind) are more likely to have suicidal thoughts or behaviors, and their risk is 1.8 times higher than that of an average family (10). The results of studies on whether adolescents in one-child or multiple-child families are more likely to develop SI are inconsistent (11), and the reasons for the inconsistent findings need to be further explored. A large number of domestic and foreign studies have reported the correlates and influences of SI in different regions and among other groups of adolescents, which cover a wide range of factors and vary considerably from study to study.

It is well known that depression and suicidal behavior are closely related. At present, the incidence of depression is showing a trend of under-aging, and the number of patients suffering from depression during adolescence is increasing year by year (12). Adolescent depression is a cause of illness and disability among adolescents aged 12-18 years, accounting for 5.7 percent (12-14 years) and 9.9 percent (15-18 years) of the global burden of disease (13). Depression in adolescents can lead to poor academic performance and impaired social functioning, increased incidence of truancy, fighting, assault, smoking, drinking, and other delinquent behaviors, as well as non-suicidal self-injury or suicidal behavior (14). Previous studies have shown that depression and SI have similar biological influences, such as genetic polymorphisms, serotonin disorders, hypothalamic-pituitary-adrenal axis dysfunction (15), and psychological factors (16). In fact, for children and adolescents, mental health is more closely related to their lives, education, and social environment. Studies have shown that early life stress is a trigger for depression in adolescents and is also closely related to suicidal behavior (14). The meta-analysis showed that individuals exposed to early life stress were 2.5 times more likely to meet the diagnostic criteria for depression in childhood or adolescence compared to those who were not exposed to early life stress (17). Specifically, behaviors that produce negative or unpleasant emotions can be adverse life events that contributing to early life stress (18). These include sexual abuse, physical abuse, and loss of a loved one at higher levels of severity, as well as lower levels of seriousness, such as poor grades, unsatisfactory interpersonal interactions, loss of a love interest, the occurrence of a life event that lowers self-esteem (e.g., being publicly humiliated), health problems, and so on (19). Verbal abuse and psychological abuse are more predictive of an individual’s negative mood, and these traumatic experiences are significantly associated with depression in adolescents (20). A study of Chinese adolescents with depression found that the top four most influential life events were found to be, in order, failure or unsatisfactory exams, heavy study load, dislike of school, pressure to go to higher education, and all related to the study (21). In a study of adolescents who had attempted suicide, it was found that all had experienced a significant life event in the year before the onset of suicidal behavior (22). Stressful life events are associated with increased SI in adolescents (23). Several studies have shown that low academic achievement, peer victimization, bullying, conflict with teachers (compared with parental conflict), and school maladjustment (including interpersonal difficulties) are risk factors for SI (8, 24). Adverse life events as a psychosocial stressor play a direct and indirect role in the development of SI and suicidal behavior. Adolescents, however, may experience more life stressors from school life, peers, or family because of their immature psychological development and lack of effective stress coping (23). Therefore, in order to improve adolescent mental health problems and prevent adolescent suicide in a timely and effective manner, it is urgent to explore further the factors associated with SI in adolescents with depression.

For many years, researchers’ interest in early life stress has focused on major traumatic events in childhood (25). Indeed, childhood trauma can be categorized into five groups: emotional abuse, physical abuse, sexual abuse, emotional neglect, and physical neglect (26). Notably, emotional trauma is more insidious and longer lasting than other trauma types, as well as being more strongly associated with depressive symptoms (27, 28). For adolescents, the family is an emotional link between parents and children. Parenting-rearing style is one of the critical environmental factors influencing adolescents’ socialization, attachment, moral reasoning, education, and problem behavior risk factors for SI (24). Parental emotional neglect and abuse during childhood have been linked to the development of insecure attachments and emotion regulation strategies, which can lead to more depressive symptoms (29). Prior research has shown that adolescents who engage in suicidal behavior tend to perceive their parents as stricter and less caring compared to adolescents who have never engaged in suicidal behavior (30, 31). Negative parenting styles (e.g., overprotection, exclusion, favoritism, punishment, and interference) increase adolescents’ risk of depression, anxiety, and suicide in adulthood (32–34). In contrast, positive parenting styles are protective factors for depression and suicide (35, 36). Currently, there are inconsistent findings on the effects of paternal or maternal parenting styles on suicidal behavior, with some studies reporting suicidal behavior associated with paternal parenting styles (37, 38) and some studies documenting harmful effects associated with maternal parenting styles (39). Few studies have investigated parenting styles in the context of Chinese culture, where authoritarian parenting is traditional. Further research on the effects of parenting styles on adolescent SI is expected to reduce adolescent suicidal behavior at its source.

Research has shown that after experiencing an adverse life event, adolescent females are more prone to negative emotions such as depression, anxiety, SI, and other high-risk factors (40, 41). Previously, we found the prevalence of SI in adolescent depression patients was 38.2%; moreover, females, less sleeping time, more learning pressure, and severe depression were associated with SI (42). Further research found that the prevalence of SI was higher in urban than in rural patients with depression; parenting rearing style may have a significant impact on the suicidal process of adolescents (43). In conjunction with existing research findings, the purpose of this study was to examine differences in (1) demographic and clinical characteristics of adolescents with depression with and without SI and (2) related factors such as parenting styles, school management, and childhood experiences.

## 2 Methods

### 2.1 Participants

A total of 1604 adolescent patients with depression aged 12-18 years were obtained in our research who belong to the First Affiliated Hospital of Harbin Medical University and the First Specialist Hospital of Harbin between May 01, 2016, and July 01, 2020, Heilongjiang province, China. The inclusion criteria included: 1) age 12-18 years, Chinese; 2) two trained clinical psychiatrists independently took the patient’s psychiatric history and diagnosed them according to the Diagnostic and Statistical Manual of Mental Disorders, Fourth Edition (DSM-IV) criteria; 3) could understand the meaning of each scale item and question and participate in clinical evaluation. The exclusion criteria included: 1) patients who suffered severe physical illness; 2) with drug and alcohol addiction; 3) refusal of patients and their guardians to sign informed consent. An initial sample of 1813 individuals was obtained in our study; 63 patients didn’t meet the diagnostic criteria for depression, 131 patients did not complete the scale, and 15 patients didn’t agree to sign the informed consent. Finally, a total of 1604 adolescents with depression were enrolled in our study. The study passed the institutional review of the First Affiliated Hospital of Harbin Medical University and the First Specialized Hospital of Harbin. Participants who agreed to participate in the survey provided a full explanation and proper consent form by the adolescent and their parents.

The sample was 12 to 18 years (M=14.86 years, SD=1.88). Of these, 723 (45.07%) were boys, and 881 (54.93%) were girls; 1193 adolescents were only children (74.38%), and 411 adolescents were not only children (25.62%). Of the total adolescents, 609 adolescents had SI (37.97%) and 995 adolescents did not have SI (62.03%).

### 2.2 Demographic characteristics

Our researchers collected clinical and demographic information including age, gender (male, female), suicide ideation (yes, no), only child (yes, no), child life (pleasant, general, unpleasant), parental rearing style (democracy, compulsory), family depression history (yes, no), school management (democracy, laissez-faire, compulsory), exercise (yes, no), sleep (less than 8 hours, more than or equal to 8 hours), smoke (yes, no), alcohol consumption (yes, no). Other information was acquired from available medical recordings and collateral resources. All information was collected from medical records and related resources. If the data were missed or ambiguous, the researchers would revisit the family or the patient’s clinical team to complete the history.

### 2.3 Clinical measures

The Chinese version of Egna Minnen Beträffande Uppfostran (EMBU) was used to assess parental rearing style. The EMBU scale was co-developed in 1980 by Carlo Perris et al. (44). The Chinese version of the scale was revised in 1993 by Yue Dongmei et al. of China Medical University (45). The Chinese version of the EMBU has high reliability and validity in evaluating parenting styles and is now widely used by Chinese adolescents to assess parenting styles. The scale contained 66 items; each item was scored from 1 to 4 (1=never, 2=occasionally, 3=often, 4=always), and it is mainly divided into two parts: paternal and maternal rearing style. There are 11 factors in this scale: paternal emotional warmth, maternal emotional warmth, paternal punishment, maternal punishment, paternal interference, maternal interference, paternal preference, maternal preference, paternal rejection, maternal rejection, and paternal overprotection. Higher-dimensional scores indicate that parents are more likely to adopt appropriate parenting styles. The EMBU has good internal consistency and construct validity. In our study, Cronbach’s alpha coefficient is 0.86.

### 2.4 Data analysis

The statistical software package used for statistical calculations in our study is the Statistical Program for Social Sciences (SPSS, version 23.0). The Kolmogorov-Smirnov test was used to verify the normal distribution of demographic variables (*P*>0.05). Descriptive statistics of adolescents with depression based on the presence or absence of SI. Frequencies and percentages were adopted for categorical variables, and mean and standard deviation (M ± SD) for continuous variables. The Pearson χ^2^ test or one-way analysis of variance (ANOVA) was used to compare demographic and clinical variables in adolescent depressed patients with and without SI, respectively. After correction for relevant variables, binary logistic regression was used to calculate the dominance ratios (ORs) for the groups with and without SI. The indicator variables corrected by Bonferroni were entered into a stepwise multiple linear regression model to explore their relationship with SI. Variables were entered into the regression model progressively from least to most significant. Covariates with a variance inflation factor (VIF) > 2.5 were excluded from the regression. The statistical test was considered with a two-tailed test, and the significance was set at 0.05 level.

## 3 Results

### 3.1 Demographic and clinical characteristics of patients

Of the 609 SI patients, the mean age of them was 15.044 ± 1.859 years; 233 were male (38.3%), 376 patients (61.7%) were female, 444 patients (72.9%) were only children, and only 26 patients (4.3%) had a family history of depression. 486 patients (79.8%) would do physical exercise, and the remaining 123 (20.2%) refused physical exercise, 378 patients (62.1%) slept less than 8 hours, 44 (7.2%) patients had been exposed to cigarettes, and 112 patients (18.4%) had a history of alcohol consumption. Of the 995 in the no-SI group, the mean age was 14.757 ± 1.900 years; 490 (49.2%) were male and 505 (50.8%) were female; 749 patients (75.3%) were only children, and 19 patients (1.9%) had a family history of depression, 859 patients (86.3%) would do physical exercise, 512 patients (51.5%) slept less than 8 hours, only 38 (3.8%) patients had been exposed to cigarettes, and 99 patients (9.9%) had a history of alcohol consumption.

### 3.2 Comparison of demographic and clinical variables between patients with and without SI

**Table 1** shows the significant differences between the patients with and without SI groups, including the following variables: gender (χ^2^ = 18.419, *p*<0.001), child life (χ^2^ = 105.461, *p*<0.001), parental rearing style (χ^2^ = 17.280, *p*<0.001), family depression history (χ^2^ = 7.715, *p*=0.005), school management (χ^2^ = 29.991, *p*<0.001), do exercise (χ^2^ = 11.893, *p*=0.001), sleep less than 8 hours (χ^2^ = 17.224, *p*<0.001), smoke (χ^2^ = 9.034, *p*=0.002), alcohol consumption (χ^2^ = 23.562, *p*<0.001), age (F= 8.779, *p*=0.003), paternal emotional warmth (F= 90.008, *p*<0.001), maternal emotional warmth (F= 80.025, *p*<0.001), paternal punishment (F= 71.249, *p*<0.001), maternal punishment (F= 73.432, *p*<0.001), paternal interference (F= 24.872, *p*<0.001), maternal interference (F= 51.594, *p*<0.001), paternal preference (F= 3.941, *p*=0.047), paternal rejection (F= 110.246, *p*<0.001), maternal rejection (F= 120.803, *p*<0.001) and paternal overprotection (F= 34.466, *p*<0.001). However, the significant differences in age, family depression history, and paternal preference did not pass the Bonferroni corrections (Bonferroni corrected *p*<0.05/22 = 0.0023). There were no significant differences in the remaining demographic and sociometric variables.

**Table 1:** Demographic and clinical variables in depressive adolescent patients with SI and without SI.

Characteristics	SI (n=609)	without SI (n=995)	F/χ^2^	*p*
Gender			18.419	<0.001
Male	233 (38.3%)	490 (49.2%)		
Female	376 (61.7%)	505 (50.8%)		
Only child			1.113	0.160
Yes	444 (72.9%)	749 (75.3%)		
No	165 (27.1%)	246 (24.7%)		
Child life			105.461	<0.001
Pleasant	310 (50.9%)	738 (74.2%)		
General	251 (41.2%)	242 (24,3%)		
Unpleasant	48 (3.9%)	15 (1.5%)		
Parental rearing style			17.280	<0.001
Democracy	365 (60%)	697 (70.1%)		
Compulsory	244 (40%)	298 (29.9%)		
Family depression history			7.715	0.005
Yes	26 (4.3%)	19 (1.9%)		
No	583 (95.7%)	976 (98.1%)		
School management			29.991	<0.001
Democracy	390 (64.0%)	757 (76.1%)		
Laissez-faire	14 (2.3%)	26 (2.6%)		
Compulsory	205 (33.7%)	212 (21.3%)		
Exercise			11.893	0.001
Yes	486 (79.8%)	859 (86.3%)		
No	123 (20.2%)	136 (13.7%)		
Sleep			17.224	<0.001
< 8 hours	378 (62.1%)	512 (51.5%)		
≥ 8 hours	231 (37.9%)	483 (48.5%)		
Smoke			9.034	0.002
Yes	44 (7.2%)	38 (3.8%)		
No	565 (92.8%)	957 (96.2%)		
Alcohol consumption			23.562	<0.001
Yes	112 (18.4%)	99 (9.9%)		
No	497 (81.6%)	896 (90.1%)		
Age	15.044±1.859	14.757±1.900	8.779	0.003
Paternal emotional warmth	46.171±13.183	52.520±12.864	90.008	<0.001
Maternal emotional warmth	49.967±13.027	55.901±12.808	80.025	<0.001
Paternal punishment	19.089±7.844	16.220±5.713	71.249	<0.001
Maternal punishment	13.956±5.311	11.864±4.360	73.432	<0.001
Paternal interference	20.394±5.328	19.138±4.613	24.872	<0.001
Maternal interference	35.102±7.925	32.327±7.238	51.594	<0.001
Paternal preference	8.406±5.010	8.936±5.298	3.941	0.047
Maternal preference	8.698±5.027	9.017±5.293	1.427	0.232
Paternal rejection	10.337±3.939	8.513±2.978	110.246	<0.001
Maternal rejection	14.301±5.473	11.644±4.151	120.803	<0.001
Paternal overprotection	10.539±3.350	9.593±2.989	34.466	<0.001

SI, suicidal ideation.

### 3.3 The risk factors of adolescent patients with SI

Based on the results of the univariate analysis of the two groups, we developed a multi-step binary logistic regression model to identify the risk factors for SI. The result was shown in **Table 2**: female (odd ratio=1.886, 95% CI=1.502–2.368, Wald χ^2^ = 29.812, *p*<0.001), sleep (odd ratio=0.798, 95% CI=0.637–0.998, Wald χ^2^ = 3.899, *p*=0.048), school management (odd ratio=1.179, 95% CI=1.041–1.336, Wald χ^2^ = 6.687, *p*=0.010), alcohol consumption (odd ratio=1.798, 95% CI=1.304–2.479, Wald χ^2^ = 12.794, *p*<0.001), child life (odd ratio=1.797, 95% CI=1.457–2.216, Wald χ^2^ = 30.059, *p*<0.001), maternal interference (odd ratio=1.032, 95% CI=1.015–1.048, Wald χ^2^ = 13.992, *p*<0.001), paternal emotional warmth (odd ratio=0.975, 95% CI=0.966–0.983, Wald χ^2^ = 31.688, *p*<0.001) and paternal rejection (odd ratio=1.102, 95% CI=1.063–1.142, Wald χ^2^ = 27.817, *p*<0.001) were dependent risk factors for SI.

**Table 2:** Risk factors related to SI by multivariate binary logistic analysis.

Variables	B	Wald χ^2^	*p*	OR	95% CI
Gender	0.634	29.812	<0.001	1.886	1.502-2.368
Sleep	-0.226	3.899	0.048	0.798	0.637-0.998
School management	0.165	6.687	0.010	1.179	1.041-1.336
Alcohol consumption	0.587	12.794	<0.001	1.798	1.304-2.479
Child life	0.586	30.059	<0.001	1.797	1.457-2.216
Maternal interference	0.031	13.992	<0.001	1.032	1.01 5-1.048
Paternal emotional warmth	-0.026	31.688	<0.001	0.975	0.966-0.983
Paternal rejection	0.097	27.817	<0.001	1.102	1.063-1.142

SI, suicidal ideation.

## 4 Discussions

Our study found that compared to adolescents without SI, those with SI had a higher proportion of females, experienced less childhood happiness, and were predominantly subjected to indulgent and authoritarian parenting styles rather than democratic ones. School management tended to be more authoritarian and less democratic. Alcohol consumption among adolescents seemed to be associated with a higher tendency towards SI. They were less inclined towards physical exercise, slept less than 8 hours, lacked parental emotional warmth, faced more frequent parental punishment, experienced higher levels of parental interference in their lives, encountered more frequent parental rejection of their requests, and were more prone to SI if excessively protected by their paternal. The logistic regression results indicate that being female, experiencing an unhappy childhood, non-democratic school management, alcohol consumption, excessive maternal interference, paternal emotional warmth, and paternal rejection play predictive roles in adolescent SI.

The findings of our research underscore the critical role of maternal interference in influencing the mental well-being of adolescents, explicitly highlighting a concerning association with SI. The identified link between maternal interference and adolescent SI underscores the profound impact of familial relationships on mental health outcomes during a crucial developmental stage. Our findings align with prior research highlighting the detrimental effects of intrusive parenting behaviors on adolescent well-being (46). Excessive maternal involvement may engender feelings of suffocation, helplessness, and a diminished sense of autonomy, creating a fertile ground for emotional distress and SI among adolescents. Several mechanisms may underpin the observed association between maternal interference and adolescent SI. Firstly, intrusive parenting may impede the development of healthy autonomy and self-efficacy, leaving adolescents feeling overwhelmed and powerless in navigating their lives (47). Secondly, constant maternal scrutiny and control may erode trust and communication within the parent-child dyad, hindering adolescents from seeking support and expressing their emotional struggles (48). Lastly, the chronic stress induced by intrusive parenting may dysregulate adolescents’ emotional responses and exacerbate vulnerability to depressive symptoms and SI (49). The identification of maternal interference as a potential risk factor for SI highlights the importance of family-based interventions in mental health programs for adolescents. It is crucial to educate parents about the potential impact of intrusive behaviors on their children’s mental health and equip them with alternative strategies for fostering autonomy and healthy communication. Moreover, mental health professionals should be vigilant in recognizing signs of excessive maternal interference during clinical assessments with adolescents. Early identification and intervention may play a pivotal role in preventing the escalation of emotional distress and SI.

Our research has found that adolescents who lack paternal emotional warmth and excessive paternal rejection are more likely to experience SI. Lack of emotional warmth and unreasonable rejection from the paternal are independent influencing factors of SI. Parenting styles significantly affect the healthy growth of individual personalities and the level of mental health. Parenting styles differed between parents, with paternal having higher rejection dimensions than maternal and the opposite being true for emotional warmth and overprotection, which may be related to the “strict paternal, kind maternal” dimension in China (50). Parental emotional warmth, particularly from paternal, is crucial in shaping adolescents’ emotional landscape and self-cognition (51). Paternals’ passionate support for adolescents positively predicts adolescent mental health more than any other factor (52). The previous study underscores the significance of parental emotional warmth in influencing adolescent mental health (53), further supporting our findings regarding the link between paternal emotional warmth and SI. The association between a lack of paternal emotional warmth and increased SI underscores the intricate link between emotional support and mental well-being. Adolescents who perceive a lack of emotional warmth from their parents may internalize feelings of rejection, hopelessness, and isolation, potentially contributing to the emergence of suicidal thoughts. In addition, the association between excessive rejection from paternal and SI underscores the negative impact of over-controlling behaviors. Adolescents experiencing unreasonable rejection may perceive heightened stress, a lack of autonomy, and a sense of inadequacy. These factors, when prolonged, can contribute to the development of SI as a coping mechanism or a perceived escape from distressing circumstances (54). The combined impact of both a lack of emotional warmth and excessive rejection from paternal intensifies the vulnerability of adolescents to SI. Interventions should consider a comprehensive approach, addressing both emotional neglect and over-control. Family-focused therapies, communication enhancement strategies, and parental education programs can be valuable components of interventions aiming to create a supportive familial environment.

Our study suggests that non-democratic school management styles can significantly impact adolescents’ mental health, particularly leading to depressive SI. Several studies have shown that the school’s management style and climate may affect adolescents’ physical and psychological health and be associated with SI (55). The authoritarian or hierarchical approaches often employed in such settings can cultivate feelings of powerlessness and disempowerment among students. This lack of agency and autonomy may exacerbate underlying mental health issues, leading to the manifestation of depressive symptoms and even SI. Conversely, schools that prioritize democratic principles, such as inclusive decision-making processes and respect for student voice, tend to foster a supportive environment that promotes emotional well-being and resilience in adolescents.

The association between an unhappy childhood and SI in adolescence underscores the profound impact early life experiences can have on mental health outcomes. Numerous studies have investigated the lasting effects of adverse childhood experiences, including unhappiness, on mental health trajectories. A study found that social and environmental adversity, self-imposed unhappiness, and externalizing disorders in childhood were associated with an increased risk of SI in adolescence and young adulthood (56). Unhappy childhood experiences are often accompanied by stress. Previous research has shown that childhood trauma increases SI in adolescents and that perceived stress mediates the relationship between childhood trauma and adolescent SI (57). Our research findings suggest recognizing the interconnectedness of early life experiences and mental health outcomes. Interventions that promote resilience, address adverse childhood experiences, and foster positive environments during formative years can play a pivotal role in preventing SI in adolescence.

In addition, female and alcohol consumption are also independent influences on SI among adolescents. Studies consistently show that females are more likely than males to experience SI. Previous studies suggested that these differences may be due to variations in coping strategies, socialization, and help-seeking behaviors between genders. Females tend to internalize distress, which may manifest as SI, while males are more likely to externalize their distress through behaviors such as substance abuse or aggression (58). Alcohol consumption, particularly heavy or problematic drinking, is strongly associated with SI among adolescents. Swahn et al. found that adolescents who reported binge drinking were more likely to report SI compared to non-binge drinkers (59). Alcohol can disinhibit individuals and impair judgment, increasing the likelihood of impulsive behavior, including suicidal thoughts. Emotional issues are also potential factors leading to SI and alcohol consumption. Borges et al. suggested that factors such as depression, anxiety, and impulsivity may mediate the relationship between alcohol use and SI among adolescents (60).

The study’s findings shed light on multiple factors associated with adolescent SI, revealing a complex interplay between parenting styles, lifestyle choices, and familial dynamics. The tendency for adolescents with SI to experience non-democratic parenting methods suggests a potential lack of communication and autonomy within the family, contributing to emotional distress (61). The reluctance or inability to engage in physical exercise and inadequate sleep among these adolescents may reflect broader mental health challenges (62). Moreover, the prevalence of parental punishment underscores potential sources of stress within the family environment. The overprotection by paternal could indicate an attempt to shield adolescents from perceived risks but may inadvertently contribute to a sense of dependency and negatively related to adolescents’ functioning (63). These results emphasize the intricate relationships between family dynamics and adolescent mental health, emphasizing the need for comprehensive interventions that address both parenting approaches and lifestyle factors to promote a supportive and nurturing environment for at-risk adolescents.

While this study provides valuable insights into the relationship between parental parenting styles and SI in adolescents with depression, several limitations should be considered. Firstly, the study’s cross-sectional design limits the ability to establish causality between variables. Longitudinal studies would be more suitable for examining how parenting styles influence the development and persistence of SI over time. Secondly, the study relied on self-report measures for assessing SI, parenting styles, and other variables. This introduces the potential for recall bias and social desirability bias, which may impact the accuracy of the data. Future studies could benefit from using multiple assessment methods, such as clinical interviews and observations, to validate the findings. Additionally, the study focused solely on adolescents with depression, which limits the generalizability of the findings to other populations. Future research should include a more diverse sample to understand better how parenting styles may interact with other factors to influence SI across different groups. In future research, we intend to incorporate more variables and influencing factors, apply multivariate analysis techniques, and further explore the predictive factors of adolescent SI. We will ensure that these results are presented in a way that enhances clarity and understanding.

In summary, our study identifies risk factors affecting adolescent SI in which parenting style plays a crucial role. Being female, experiencing an unhappy childhood, non-democratic school management, alcohol consumption, excessive maternal interference, lack of paternal emotional warmth, and paternal rejection were predictive of adolescent SI. Clinicians are advised to prioritize female adolescents as well as adolescents who drink alcohol. Once they are found to have SI, they should promptly inform their guardians to strengthen 24-hour care, stay away from suicidal tools or places, and actively carry out psychopharmacological interventions. Advocate for the democratization of school management style and adopt regular mental health surveys. Psychological counseling hotlines should be provided, and professional psychotherapists should be hired to assist in monitoring the mental health of schoolchildren when necessary. Encourage parents to help their children plan their childhood experiences, learn proper parenting styles, and strengthen parent-child education. Specifically, mothers should reduce their interference with their children and give adolescents appropriate autonomy; fathers should provide more love and emotional warmth and minimize rejection, and it is important to satisfy and explain adolescents’ requests reasonably. Due to methodological limitations, future longitudinal or retrospective cohort studies are recommended to determine further the causal relationship between SI and related factors and to investigate the association between related comorbidities and parenting styles. Although these results remain to be studied in depth, focusing on parenting styles to reduce SI is essential to promote the health and well-being of adolescents.

## Data Availability

The original contributions presented in the study are included in the article/**Supplementary Material**. Further inquiries can be directed to the corresponding authors.
